# Theoretical Mechanistic and Kinetic Studies on Homogeneous Gas-Phase Formation of Polychlorinated Naphthalene from 2-Chlorophenol as Forerunner

**DOI:** 10.3390/ijms161025641

**Published:** 2015-10-26

**Authors:** Fei Xu, Ruiming Zhang, Yunfeng Li, Qingzhu Zhang, Wenxing Wang

**Affiliations:** Environment Research Institute, Shandong University, Jinan 250100, China; E-Mails: zrm0118@163.com (R.Z.); liyfeng1028@gmail.com (Y.L.); zqz@sdu.edu.cn (Q.Z.); wxwang@sdu.edu.cn (W.W.)

**Keywords:** 2-chlorophenol, reaction mechanism, rate constants, formation of polychlorinated naphthalene, and theoretical mechanistic and kinetic study

## Abstract

Polychlorinated naphthalenes (PCNs) are dioxins-like compounds and are formed along with polychlorinated dibenzo-*p*-dioxins (PCDDs) and polychlorinated dibenzofurans (PCDFs) in thermal and combustion procedures. Chlorophenols (CPs) are the most important forerunners of PCNs. A comprehensive comprehension of PCN formation procedure from CPs is a precondition for reducing the discharge of PCNs. Experiments on the formation of PCNs from CPs have been hindered by PCN toxicity and short of precise detection methods for active intermediate radicals. In this work, PCN formation mechanism in gas-phase condition from 2-chlorophenol (2-CP) as forerunner was studied by quantum chemistry calculations. Numbers of energetically advantaged formation routes were proposed. The rate constants of key elementary steps were calculated over 600–1200 K using canonical variational transition-state theory (CVT) with small curvature tunneling contribution (SCT) method. This study illustrates formation of PCNs with one chlorine atom loss from 2-CP is preferred over that without chlorine atom loss. In comparison with formation of PCDFs from 2-CP, PCN products are less chlorinated and have lower formation potential.

## 1. Introduction

Polychlorinated naphthalenes (PCNs) show similar geochemical characters, biological properties, structures and toxicities to polychlorinated dibenzo-*p*-dioxins and dibenzofurans (PCDD/Fs). The PCN toxicity is analogous with PCDD/F in some human serum samples [1] and even higher than that of PCDD/Fs in some typical locations [2,3]. Similar to other persistent organic pollutants (POPs), PCNs are ubiquitous contaminants found in air, snow, sediments and biota, even in the polar environments [4]. PCNs are candidate POPs according to the POPs Protocol of the United Nations Economic Commission for Europe [5], and have recently been proposed for listing under the priority controlling roster of the Stockholm Convention on POPs [6]. Hence, activities for controlling and reducing PCN emissions may be obligatory in the near future.

PCNs have been commercially synthesized since the 1910s. Besides historical residue, the key origins of PCNs currently are inadvertent emission from thermal procedures and incomplete combustion processes as outgrowths, along with polychlorinated biphenyl (PCBs), PCDDs and PCDFs [7,8,9,10,11,12,13,14,15]. PCNs from waste incinerator were tested at analogous concentrations as the total PCDD/Fs [16]. In addition, several studies revealed a considerably stronger relationship of PCN and PCDF mass concentrations or isomer distributions in thermal processes than that of PCN and PCDD, suggesting that the formation pathways of PCN and PCDF are more similar than that of PCN and PCDD [17,18,19,20,21,22,23].

Several PCN formation mechanisms have been proposed [10,11,12,13,17,19,21,22,23,24,25,26,27], including chlorination of unsubstituted naphthalene [24,25], *de novo* synthesis from polycyclic aromatic hydrocarbon (PAHs) [17,19], hydrogen abstraction acetylene addition mechanisms [26,27], and chlorophenols (CPs) condensation [10,11,12,13,21,22,23] via gas-phase homogeneous or metal-mediated heterogeneous reactions. Among the various forerunners, CPs are the main forerunners in the formation of PCNs. Numbers of experimental studies have demonstrated that PCN form in gas-phase pyrolysis and oxidation from CPs along with PCDD/Fs [10,11,12,13,21,22,23]. In thermal and combustion procedures, CPs can produce chlorophenoxy radicals (CPRs) by abandoning a hydroxy-hydrogen. Based on some lab results, Kim raised an exhaustive PCN formation mechanism [21,22,23]; in this mechanism, PCNs are produced via C–C connection on unchlorinated *ortho* CPR positions, forming a chlorinated *o*,*o*′-dihydroxybiphenyl (chloro-DOHB) intermediate [21,22,23]. For one thing, chloro-DOHB may form PCDFs via H migration, isomerization and H_2_O leave, which we previously studied [28,29,30,31]. For another, chloro-DOHB can form chlorinated dihydrofulvalene by two carbon monoxide elimination steps and two ring close reactions, followed by PCN formation via routes similar to the mechanism proposed by Melius [21,22,23,32]. This mechanism can explain the more similar formation connection of PCN with PCDF than PCDD in thermal processes.

However, the specific PCN formation mechanism is still uncertain. The predicted PCN formation mechanism by Kim cannot explain some of the experimental observations [21,22]. Firstly, Kim could not explain his experimental observation wherein the yield of monochlorinated naphthalenes (MCNs) is significantly higher than that of dichlorinated naphthalenes (DCNs) from 2-CP, according to the similar formation pathway number of MCNs and DCNs in his proposed mechanism [21,22]. Secondly, the amounts of 1,8-DCN detected in the experiment were rare, which is comparable with the amounts of 1,5-/1,6-/1,7-DCNs in his mechanism [21,22]. Moreover, Kim inferred that Cl shift in cyclopentadiene ring of dihydrofulvalene [21,22] may be another important reaction route, leading to the formation of additional PCN isomers. As reported, the 1,5-sigmatropic shift of Cl is similar to that of H in chlorinated cyclopentadiene [33]. Besides, H/Cl atom may be directly abstracted. These two possible reactions need to be further studied. Furthermore, another PCN formation study from 2-CP comes from Yang [11], and part of his results are not consistent with Kim’s observation. For example, Yang found that 2-CP produced more 2-MCN than 1-MCN [11], whereas Kim observed that 2-CP produced mostly 1-MCN. To solve all the contradictions above, new PCN formation pathways and more detailed mechanisms from 2-CP need to be proposed.

Quantum chemical calculation can be used to research highly toxic compounds, predict the feasibility of a reaction route and confirm the priority of the products. Kim also repeatedly mentioned in his experimental study that a detailed computational study is needed to elucidate PCN formation [21,22]. In this study, we present an overall density functional theory (DFT) research of PCN gas-phase formations from 2-CP. Secondly, rate constants for the major elementary reactions over 600–1200 K were evaluated. All possible formation pathways involved in PCN formation from 2-CP as forerunner were studied. Some energetically preferred routes were proposed to parallel the formation possibility of different PCN products and explain experimental observations.

## 2. Results and Discussion

### 2.1. 2-Chlorophenoxy Radical Formation from 2-Chlorophenols

**Figure 1:**
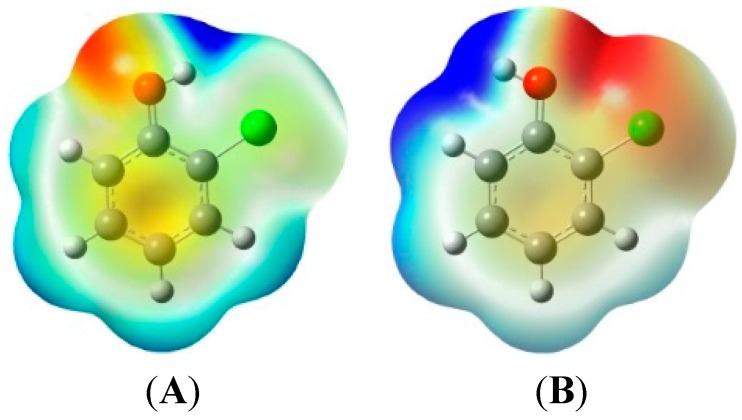
The electron densities of *syn* and *anti* 2-CP. (**A**) *syn* 2-CP; (**B**) *anti* 2-CP. 2-CP: 2-chlorophenol.

CPs are the most crucial forerunners in gas-phase PCN formation [10,11,12,13,21,22,23]. For 2-CP, there are two structure conformers (*syn* 2-CP and *anti* 2-CP). The *syn* 2-CP has the hydroxyl-hydrogen toward the closest neighboring Cl atom on the benzene ring and the *anti* 2-CP has the hydroxyl-hydrogen toward the closest neighboring H atom on the benzene ring. The *syn* conformer has an intramolecular H–Cl hydrogen bond. The bond distance of *syn* 2-CP (0.959 Å) is longer than that of *anti* 2-CP (0.955 Å). This indicates that the O–H bond in *syn* 2-CP is weaker than that in *anti* 2-CP, and the phenoxyl-hydrogen in *anti* 2-CP are more easily to be abstracted than *syn* 2-CP. However, the electronic energy of *syn* 2-CP is lower by about 3 kcal/mol than that of the *anti* 2-CP. This can be explained by the HOMO-LOMO energy gap in *syn* and *anti* 2-CPs. The HOMO-LOMO energy gap of *syn* 2-CP (0.27823 ev) is larger than that of *anti* 2-CP (0.27542 ev). The electron densities of *syn* and *anti* 2-CPs are shown as follows in Figure 1. Throughout this paper, 2-CP denotes the syn conformer.

Dimerization of CPRs to form chloro-DOHB intermediate is crucial in formation from CPs [10,11,12,13,21,22,23]. Thus, the initiative and important step is the CPR formation from CPs. In thermal and combustion procedures, CPs can abandon hydroxy-hydrogen to produce CPRs via O–H bond direct break or being abstracted by OH, H, Cl and O(^3^P). The formations of 2-CPRs from 2-CP have been investigated in detail in our previous studies [28,34,35].

### 2.2. Chloro-Dihydrofulvalene Production from Dimerization of 2-Chlorophenoxy Radicals

**Figure 2:**
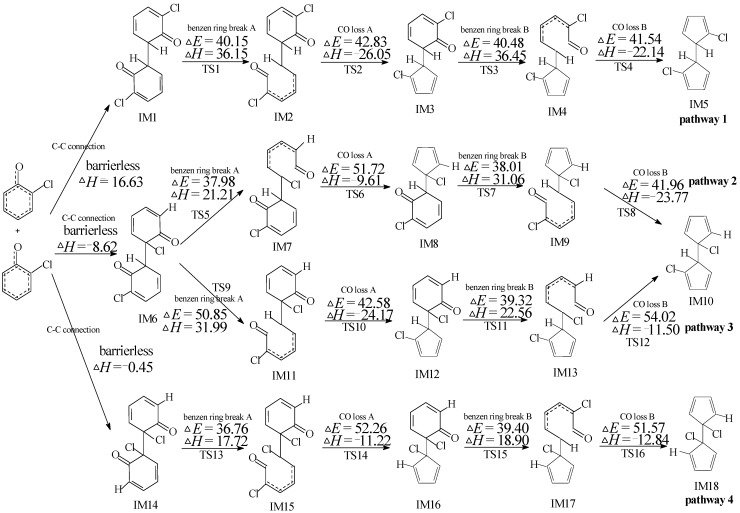
Chlorinated dihydrofulvalene formation routes embedded with the potential barriers Δ*E* (in kcal/mol) and reaction heats Δ*H* (in kcal/mol) from the 2-CP as forerunner at the MPWB1K/aug-cc-pVTZ//MPWB1K/6-31+G(d,p) level. Δ*H* is calculated at 0 K. IM: intermediate; TS: transition state.

As shown in Figure 2, four probable formation routes (pathways 1–4) to form three chloro-dihydrofulvalenes are proposed from the coupling of 2-CPRs. “IM” is the abbreviation of “intermediate”, and “TS” is the abbreviation of “transition state”. All chloro-dihydrofulvalene formation pathways start from C–C connection, followed by two benzen ring break steps (benzen ring break A and benzen ring break B) and two CO loss steps (CO loss A and CO loss B). The dimerization of 2-CPRs is strongly exothermic and barrierless. The first or second CO elimination step is a synergetic reaction together with the formation of a five-member ring. The ranking for the exothermic values of the three C–C connection steps is as follows: CH/CH (in pathway 1) > CH/CCl (in pathways 2 and 3) > CCl/CCl (in pathway 4), owing to the steric effect [28]. In addition, the largest barrier in pathway 1 is 42.83 kcal/mol, pathway 2 has one >50 kcal/mol barrier and pathway 3 and 4 have two steps with >50 kcal/mol barrier. Thus, considering the two aspects, pathway 1 is the most reasonable, and then pathway 2, resulting the formation of IM5 and IM10. Pathway 3 and pathway 4 are energetically infeasible.

### 2.3. PCN Formation from the Following Reactions of IM5

**Figure 3:**
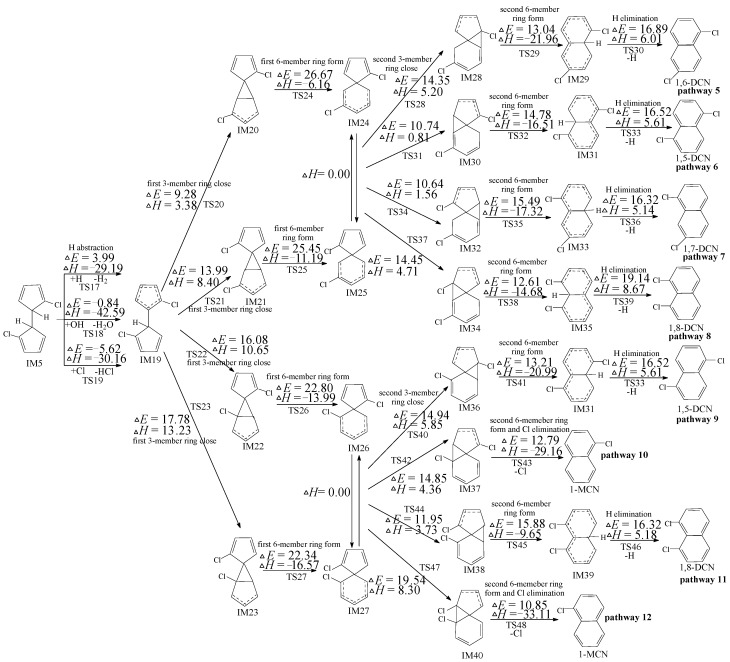
PCN formation routes embedded with the potential barriers Δ*E* (in kcal/mol) and reaction heats Δ*H* (in kcal/mol) from IM5 at the MPWB1K/aug-cc-pVTZ//MPWB1K/6-31+G(d,p) level. Δ*H* is calculated at 0 K. PCN: Polychlorinated naphthalene; MCN: monochlorinated naphthalene; DCN: dichlorinated naphthalene.

In Figure 3, eight probable reaction routes are presented for PCN formation from the following reactions of IM5. IM24 and IM25 are enantiomers that form the identical following intermediate (IM28, IM30, IM32 and IM34). For example, “1-MCN” means that the H atom in C_1_ of naphthalene is substituted with Cl atom. “1,6-DCN” means that the H atom in both C_1_ and C_6_ of naphthalene are substituted with two Cl atoms. In Figure 3, pathways 5, 6, 7, 8, 9 and 11 are similar, containing the subsequent six elementary reactions. The first six-member ring form reaction is the rate controlling reaction. This mechanism is similar to the mechanism proposed by Melius of naphthalene from 9,10-dihydrofulvalene [32]. Pathways 10 and 12 are homologous and they embody five elementary reactions with the last synergetic reaction. The rate controlling reaction for pathways 10 and 12 remain in the third step similarly with pathways 5, 6, 7, 8, 9 and 11.

In Figure 3, pathways 9–12 possess the identical rate controlling reactions, which occur via lower barriers than those of pathways 5–8. In addition, pathways 10 and 12 occur via one step less than pathways 5, 6, 7, 8, 9 and 11, *i.e.*, pathways terminated of Cl loss is preferred than those terminated of H loss. Thus, pathways 10 and 12 are energetically more feasible, leading to the formation of 1-MCN, which supports the experimental result by Kim wherein 1-MCN is the main MCN from 2-CP as forerunner [21,22], and opposes the conclusion by Yang that 2-MCN is much easier to form than 1-MCN [21]. This also successfully explains the lab results by both Yang and Kim wherein MCN formation possibility with one Cl leave is larger than DCN formation possibility without Cl leave [11,21,22]. Similar reactions can be obtained from polychlorinated dibenzo-*p*-dioxins (PCDD) formation from chlorophenols (CPs) and polychlorinated thianthrene (PCTA) formation from chlorothiophenols (CTPs), which show that pathways formation possibility Cl loss remains more feasible than that terminated of H loss [28,29,30,31,36,37].

From Figure 3, 4 DCN congeners (1,5-/1,6-/1,7-/1,8-DCNs) from eight pathways (pathways 5, 6, 7, 8, 9 and 11) are proposed. Among them, pathways 9 and 11 occur via the same enantiomer intermediate IM26/IM27 with pathways 10 and 12, and directly compete with the energetically preferred pathways 10 and 12. Thus, pathways 9 and 11 are energetically unfeasible than other DCN formation pathways (pathways 5–8). Furthermore, pathways 5–8 occur via the same enantiomer intermediate IM24/IM25, and the distinctions of pathways 5–8 are in the ending three elementary reactions. H loss step in pathway 8 contains the largest potential barrier and is most endoergic of the three elementary reactions; thus, pathway 8 is not thermodynamically favored for DCN formation compared with pathways 5–7. In summary, the two 1,8-DCN formation pathways (pathways 8 and 11) are energetically unfavorable. This give a reasonable explanation that almost no 1,8-DCN were experimentally obtained from 2-CP. Major DCNs are produced via pathway 5, 6 and 7, resulting in 1,5-/1,6-/1,7-DCNs, which was also observed in Kim’s experiment [21,22].

**Figure 4:**
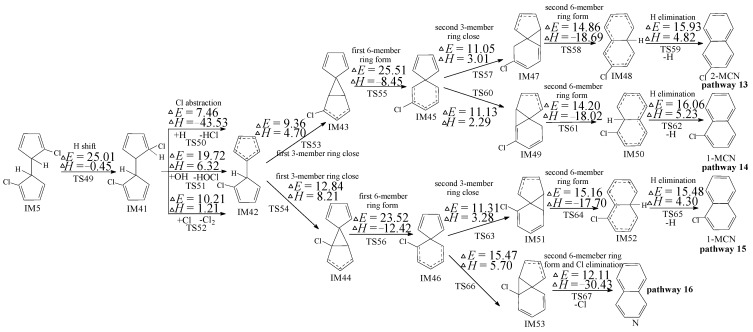
PCN formation routes from IM5 proposed by Kim [21,22], starting with *H*-shift step. These routes are embedded with the potential barriers Δ*E* (in kcal/mol) and reaction heats Δ*H* (in kcal/mol) at the MPWB1K/aug-cc-pVTZ//MPWB1K/6-31+G(d,p) level. Δ*H* is calculated at 0 K.

The subsequent reactions of IM5 were inferred by Kim according to the experimental results [21,22], containing the 1,5-sigmatropic H shift step as the following step of IM5 instead of the H abstraction step. For comparison, the subsequent reaction of IM5 proposed by Kim was also studied using quantum chemistry as shown in pathways 13–16 of Figure 4. In Figure 4, “N” is the abbreviation of “naphthalene”. In our mechanism shown in Figure 3, H is directly abstracted by OH, H, and Cl, whereas, as shown in Figure 4, H migration occur first. However, the H migration step needs via a lager potential barrier (25.01 kcal/mol). Therefore, our H direct abstraction mechanism (pathways 5–12) shown in Figure 3 is preferred than H shift mechanism (pathways 13–16) proposed by Kim shown in Figure 4.

### 2.4. PCN Formation from the Following Reactions of IM10

**Figure 5:**
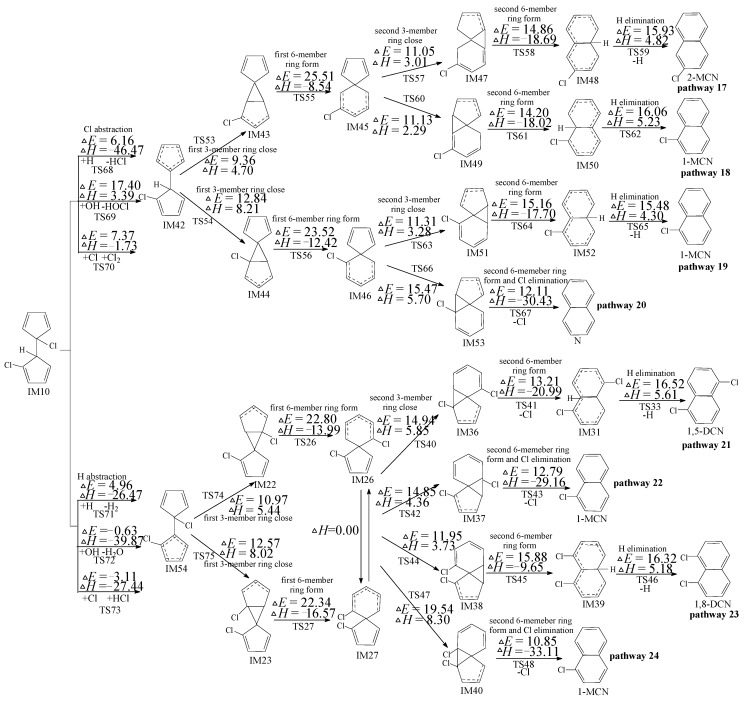
PCN formation routes embedded with the potential barriers Δ*E* (in kcal/mol) and reaction heats Δ*H* (in kcal/mol) from IM10 at the MPWB1K/aug-cc-pVTZ//MPWB1K/6-31+G(d,p) level. Δ*H* is calculated at 0 K.

In Figure 5, eight probable reaction pathways (pathways 17–24) are raised for the following reactions of IM10 to form PCNs. Pathways 17, 18, 19, 21 and 23 are alike, and pathways 20, 22 and 24 are similar. From Figure 5, IM10 can form PCNs via pathways 17–20 initiated by Cl abstraction step or pathways 21–24 initiated by H abstraction step. In the Cl abstraction pathways, pathways 17 and 18 possess the identical rate controlling reaction (25.51 kcal/mol barriers), and pathways 19 and 20 possess the same rate controlling reactions (23.52 kcal/mol barriers). The rate controlling step of pathway 19 and 20 are cross lower barriers than those of pathways 17 and 18. In addition, pathway 20 has one step less than pathways 17–19, *i.e.*, pathways terminated of Cl loss prefer over those terminated of H loss. Thus, pathway 20 is favored over pathways 17–19, leading to the formation of naphthalene (N). N is the main product of Cl abstraction pathways, *i.e.*, pathways 17–20. In the H abstraction pathways (pathways 21–24), IM26 and IM27 are enantiomers that form the uniform subsequent intermediate (IM36, IM37, IM38 and IM40), via two rate-determining steps with similar potential barrier and reaction heats. However, pathways 22 and 24 with Cl of have one step less than pathways 21 and 23 with H loss. Thus, pathways 22 and 24 are preferred, *i.e.*, 1-MCN is the main product of H abstraction pathways (pathways 21–24). This reconfirm the conclusion above and Kim’s experiment results that 1-MCN is the main MCN product from 2-CP as forerunner.

It is important to parallel the formation potential of N from Cl abstraction pathways and that of 1-MCN from H abstraction pathways. From Figure 5, the intermediate IM42 can be regarded as a prestructure for N. IM54 is a prestructure for 1-MCN. As shown in Figure 5, the formation of IM54 from IM10 abstracted by OH, H or Cl occur via lower potential barrier than that of IM42, respectively. Furthermore, the rate controlling reaction of 1-MCN formation has lower barrier compared with that of N formation. Thus, 1-MCN formation is preferred over N formation. In addition, the subsequent reactions of IM5 also produce 1-MCN, which greatly increase the yield of 1-MCN. However, both Kim and Yang observed more N formation than 1-MCN from 2-CP as forerunner in their experiments [11,21,22]. More N may be produced via crossed coupling of phenoxy radical with 2-CPR or self-coupling of phenoxy radicals. In the Kim’s experiments, phenol was largely present [21,22].

Similar as IM5, IM10 may occur in the 1,5-sigmatropic H migration step, as shown in pathways 25 and 26 of Figure S1. The direct H abstraction mechanisms (pathways 21–24) demonstrated in Figure 5 are preferred to the H migration process (pathways 25 and 26) shown in Figure S1. Moreover, Kim proposed that Cl migration to *ortho*-carbon before *ortho*-carbon H abstraction may be another possible reaction sequence of IM10, as shown in pathways 27–34 in Figure S1. In comparison to the direct Cl abstraction pathways (pathways 17–20) in Figure 5, the Cl shift step is also via a larger barrier (27.99 kcal/mol). Therefore, the direct Cl abstraction pathways 17–20 shown in Figure 5 are also preferred over the 1,5-sigmatropic Cl migration pathways 27–34 proposed by Kim shown in Figure S1.

### 2.5. Formation Comparison PCNs and PCDFs from 2-Chlorophenol

Our previous studies showed the detailed PCDF formations pathways from 2-CP with the same carbon-carbon coupling DOHB intermediate as PCN formation [28]. Formation of PCDFs needs only five elementary steps, and rate controlling step has the barrier <30 kcal/mol [28]. In this study, PCN formation needs ten or eleven steps, and the rate controlling reaction has the potential barrier >40 kcal/mol. Thus, the yield of total PCNs is much lower than that of PCDFs, which agree well with experimental observations [11,12,13,21,22,23]. Moreover, a comparison of the distributions of PCDFs and PCNs products reveals that 4,6-DCDF is the most product [28], whereas 1-MCN is the major PCN product from 2-CP. This result means PCNs tend to form Cl loss isomers, whereas PCDF formation does not cross the Cl loss step.

### 2.6. Rate Constant Calculations

In this section, canonical variational transition state theory (CVT) with small-curvature tunneling (SCT) contribution were used to calculate the rate constants of the key elementary reactions for the formation of PCNs from 2-CP over 600–1200 K. The CVT/SCT values are expressed in the Arrhenius form as shown in Table 1. The branching radios of the branching reactions at 1000 K are listed in brackets after the reactions in Table 1. A comparison of the rate-controlling reaction of PCDFs and PCNs from CPs would be interesting for further investigation of the formation yields of PCDFs and PCNs [28]. As for the route of CH/CH coupling of 2-CPRs, from our original paper, the rate-controlling reaction of PCDFs from 2-CPRs is a ring close step with the Arrhenius form (2.11 × 10^12^) exp (−14,722.61/*T*) [28], and the rate-controlling reaction of PCNs from 2-CPRs is CO loss step of IM2 → IM3 + CO via TS2 with the Arrhenius form (2.32 × 10^11^) exp (−21,473.60/*T*). At 1000 K, the value of ring close step involved in the PCDF formation is 8.52 × 10^5^ cm^3^·molecule^−1^·s^−1^ [28], which is larger than that of 1.54 × 10^2^ cm^3^·molecule^−1^·s^−1^ of IM2 → IM3 + CO. This result could explain the experimental observation in which the yield of PCNs is considerably less than that of PCDFs.

**Figure 6:**
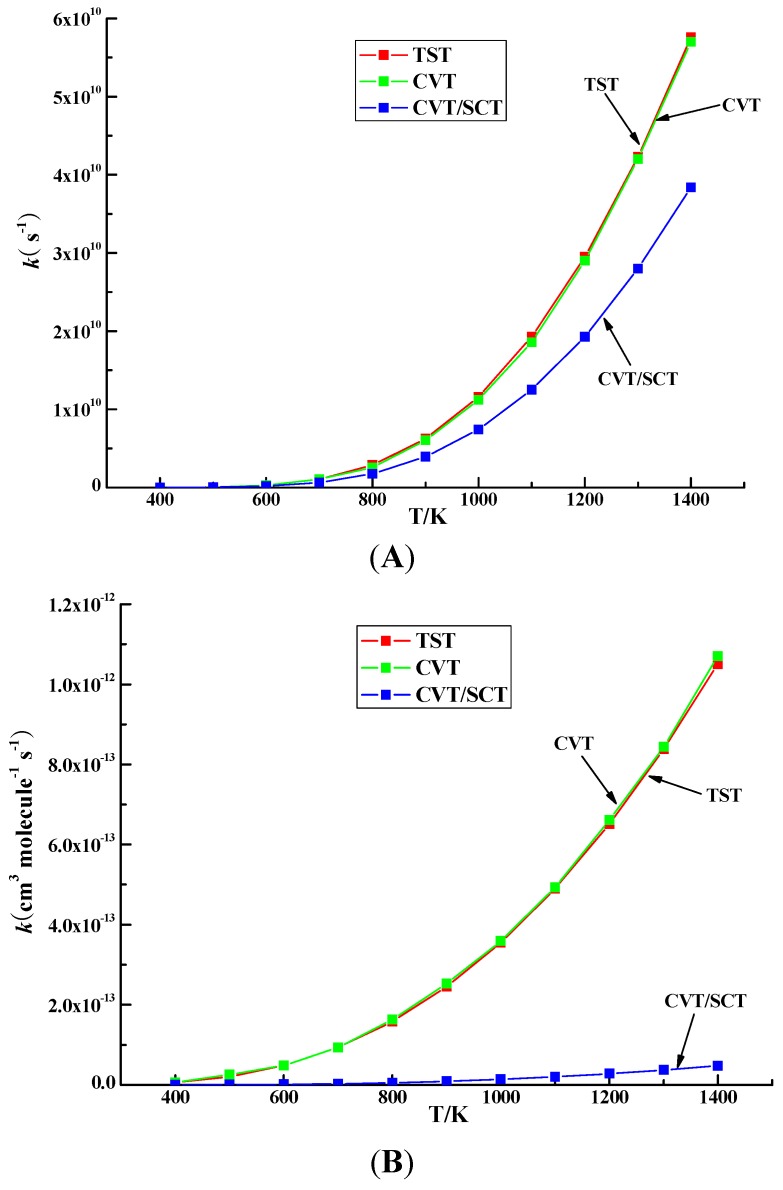
Arrhenius plot of the CVT/SCT rate constants in the temperature range of 600–1200 K for reaction of (**A**) IM24/IM25 → IM32 via TS34 and (**B**) IM5 + H → IM19 + H_2_ via TS17. CVT: canonical variational transition-state theory; SCT: small curvature tunneling contribution.

In this study, there exit two reaction modes: unimolecular reactions (with the unit of rate constant s^−1^) and bimolecular reactions (with the unit of rate constant cm^3^·molecule^−1^·s^−1^). We take IM24/IM25 → IM32 via TS34 and IM5 + H → IM19 + H_2_ via TS17 and as the representative reaction for monomolecular reactions and bimolecular reactions. The Arrhenius plot of the CVT/SCT values together with TST and CVT values over 600–1200 K for reaction of IM24/IM25 → IM32 via TS34 and IM5 + H → IM19 + H_2_ via TS17 are shown in Figure 6. For the reaction of IM24/IM25 → IM32 via TS34, in 600–800 K, the TST values and CVT/SCT values are nearly coincident, which means the tunneling effect is small. However, with temperature increasing, the differences between TST and CVT/SCT values become larger. For the reaction of IM5 + H → IM19 + H_2_ via TS17, as the temperature increases, the differences between TST values and CVT/SCT values is larger over the whole studied 600−1200 K. The gap of TST values and CVT/SCT values for IM5 + H → IM19 + H_2_ via TS17 grows faster than that of IM24/IM25 → IM32 via TS34. For example, at 1200 K, the ratio of TST value and CVT/SCT value for IM24/IM25 → IM32 via TS34 via TS17 is 1.5, whereas the ratio is 23.3 for IM5 + H → IM19 + H_2_ via TS17. The latter is 15.5 times larger than the former.

**Table 1:** Arrhenius formulas for crucial elementary reactions involved in the formation of PCNs from the 2-CP forerunner over the temperature range of 600–1200 K (units are s^−1^ and cm^3^·molecule^−1^·s^−1^ for unimolecular and bimolecular reactions, respectively) based on the MPWB1K/aug-cc-pVTZ// MPWB1K/6-31+G(d,p) energies.

Reactions	Arrhenius Formulas
IM1 → IM2 via TS1	*k* (*T*) = (3.12 × 10^12^) exp (−21,500.36/*T*)
IM2 → IM3 + CO via TS2	*k* (*T*) = (2.32 × 10^11^) exp (−21,473.60/*T*)
IM3 → IM4 via TS3	*k* (*T*) = (1.21 × 10^13^) exp (−21,309.58/*T*)
IM4 → IM5 + CO via TS4	*k* (*T*) = (1.17 × 10^11^) exp (−21,746.24/*T*)
IM6 → IM7 via TS5	*k* (*T*) = (1.94 × 10^13^) exp (−20,475.72/*T*)
IM7 → IM8 + CO TS6	*k* (*T*) = (2.41 × 10^11^) exp (−24,764.15/*T*)
IM8 → IM9 via TS7	*k* (*T*) = (5.43 × 10^13^) exp (−20,072.04/*T*)
IM9 → IM10 + CO via TS8	*k* (*T*) = (3.33 × 10^11^) exp (−21,413.18/*T*)
IM6 → IM11 via TS9	*k* (*T*) = (2.14 × 10^13^) exp (−26,398.65/*T*)
IM11 → IM12 + CO via TS10	*k* (*T*) = (6.17 × 10^11^) exp (−21,840.02/*T*)
IM12 → IM13 via TS11	*k* (*T*) = (1.37 × 10^13^) exp (−39,956.40/*T*)
IM13 → IM10 + CO via TS12	*k* (*T*) = (3.70 × 10^11^) exp (−28,457.34/*T*)
IM5 + H → IM19 + H_2_ via TS17	*k* (*T*) = (6.47 × 10^−13^) exp (−3826.40/*T*)
IM19 → IM20 via TS20 (0.93)	*k* (*T*) = (5.80 × 10^12^) exp (−4953.85/*T*)
IM19 → IM21 via TS21 (0.04)	*k* (*T*) = (2.76 × 10^12^) exp (−7312.34/*T*)
IM19 → IM22 via TS22 (0.02)	*k* (*T*) = (3.09 × 10^12^) exp (−8295.86/*T*)
IM19 → IM23 via TS23 (0.01)	*k* (*T*) = (4.04 × 10^12^) exp (−9165.93/*T*)
IM22 → IM26 via TS26	*k* (*T*) = (1.70 × 10^13^) exp (−11,865.38/*T*)
IM23 → IM27 via TS27	*k* (*T*) = (1.82 × 10^13^) exp (−11,673.73/*T*)
IM24/IM25 → IM28 via TS28 (0.06)	*k* (*T*) = (1.77 × 10^12^) exp (−7432.09/*T*)
IM24/IM25 → IM30 via TS31 (0.50)	*k* (*T*) = (2.48 × 10^12^) exp (−5572.69/*T*)
IM24/IM25 → IM32 via TS34 (0.40)	*k* (*T*) = (2.24 × 10^12^) exp (−5708.95/*T*)
IM24/IM25 → IM34 via TS37 (0.11)	*k* (*T*) = (1.61 × 10^12^) exp (−7509.02/*T*)
IM26/IM27 → IM36 via TS40 (0.11)	*k* (*T*) = (1.98 × 10^12^) exp (−7696.65/*T*)
IM26/IM25 → IM37 via TS42 (0.18)	*k* (*T*) = (3.29 × 10^12^) exp (−7701.35/*T*)
IM37 → 1-MCN + Cl via TS43	*k* (*T*) = (2.03 × 10^13^) exp (−6825.08/*T*)
IM26/IM27 → IM38 via TS44 (0.69)	*k* (*T*) = (2.77 × 10^12^) exp (−6154.47/*T*)
IM26/IM27 → IM40 via TS47 (0.03)	*k* (*T*) = (7.40 × 10^11^) exp (−8061.95/*T*)
IM40 → 1-MCN + Cl via TS48	*k* (*T*) = (3.50 × 10^13^) exp (−5895.30/*T*)
IM10 + H → IM42 + HCl via TS68	*k* (*T*) = (1.56 × 10^−11^) exp (−4151.85/*T*)
IM10 + OH → IM42 + H_2_O via TS69	*k* (*T*) = (6.16 × 10^−12^) exp (−10,425.51/*T*)
IM10 + Cl → IM42 + Cl_2_ via TS70	*k* (*T*) = (1.04 × 10^−10^) exp (−5075.97/*T*)
IM42 → IM43 via TS53 (0.92)	*k* (*T*) = (1.98 × 10^12^) exp (−7696.65/*T*)
IM42 → IM44 via TS54 (0.08)	*k* (*T*) = (1.09 × 10^12^) exp (−6633.06/*T*)
IM44 → IM46 via TS56	*k* (*T*) = (2.16 × 10^13^) exp (−13,348.67/*T*)
IM45 → IM47 via TS57 (0.51)	*k* (*T*) = (2.90 × 10^12^) exp (−5798.88/*T*)
IM45 → IM49 via TS60 (0.49)	*k* (*T*) = (2.75 × 10^12^) exp (−5797.29/*T*)
IM46 → IM51 via TS63 (0.91)	*k* (*T*) = (3.40 × 10^12^) exp (−5853.60/*T*)
IM46 → IM53 via TS66 (0.09)	*k* (*T*) = (2.94 × 10^12^) exp (−8057.90/*T*)
IM53 → N + Cl via TS67	*k* (*T*) = (1.93 × 10^13^) exp (−6496.42/*T*)
IM10 + H → IM54 + H_2_ via TS71	*k* (*T*) = (6.32 × 10^−11^) exp (−3311.61/*T*)
IM54 → IM22 via TS74 (0.62)	*k* (*T*) = (2.34 × 10^12^) exp (−5539.60/*T*)
IM54 → IM23 via TS75 (0.38)	*k* (*T*) = (3.71 × 10^12^) exp (−6473.85/*T*)

PCN: Polychlorinated naphthalene; 2-CP: 2-chlorophenol; IM: intermediate; TS: transition state.

## 3. Experimental Section

### 3.1. Density Functional Theory

Gaussian 09 program (Wallingford, CT, USA) was employed for all the electronic structure, frequency and energy calculations for reactants, intermediates, transition states and products [38]. The MPWB1K function was selected in this research, which is widely used in the thermodynamics, kinetic, and weak interaction calculations [39]. Structure optimization was calculated using the MPWB1K/6-31+G(d,p) standard. Frequency calculations were performed using 6-31+G(d,p) basis set to distinguish the transition states (with one and only one imaginary frequency) and stable intermediates (with no imaginary frequency). The intrinsic reaction coordinates (IRC) were calculated to ensure that transition states link the correct reactions and products [40]. The energy calculations of the various species were employed using two more flexible basis sets, aug-cc-pVTZ and 6-311+G(3df,2p). Figures and Tables based at the MPWB1K/aug-cc-pVTZ level are revealed in Table 1 and Figure 2, Figure 3, Figure 4 and Figure 5 in the manuscript and Table S1 and Table S2 and Figure S1 in Supplementary Materials. Figures and Tables based on the at the MPWB1K/6-311+G(3df,2p) level are demonstrated in Supplementary Materials Table S3 and Figures S2–S6.

### 3.2. Kinetic Calculation

Rate constants of major elementary reaction at 600–1200 K were calculated using CVT/SCT method [41,42,43,44] on Polyrate 9.7 software (University of Minnesota, Minneapolis, MN, USA) [45]. The SRANGE, which is needed to specify the limits on the reaction coordinate, were selected from −1.5 to 1.5. The SSTEP, which is a variable keyword in Polyrate 9.7 program (University of Minnesota, Minneapolis, MN, USA) that specifies the step size along the mass-scaled MEP, were confirmed as 0.05. This method has been comprehensively used in our previous papers on the rate constants of PCDD/F formation and degradation [28,29,30,31,46,47,48].

## 4. Conclusions

(1) CPRs coupling consists of three types, and the formation ranking for these three types is CH/CH > CH/CCl > CCl/CCl.

(2) Pathways terminated with Cl elimination (pathways for the MCN formation) prefer over those terminated with H elimination (pathways for the DCN formation).

(3) The main MCN product is 1-MCN, and the main DCN products are 1,5-/1,6-/1,7-DCNs.

(4) The first step of PCN formation from chloro-dihydrofulvalenes is *ortho*-carbon H or Cl direct abstraction by H, OH or Cl radicals, not via 1,5-sigmatropic H/Cl shift before.

## Supplementary Materials

PCN formation routes from IM10 proposed by Kim. Imaginary frequencies, total energies, zero point energies, thermal correction to energy, thermal correction to enthalpy, thermal correction to gibbs free energy at the MPWB1K/aug-cc-pVTZ//MPWB1K/6-31+G(d,p) level, for the transition states involved in the PCN formation from 2-CP. The potential barriers with ZPE correction (Δ*E*) and the reaction heats with ZPE correction (Δ*H*, 0 K), potential barriers with thermal correction to energy (Δ*Ea*) and the reaction heats with thermal correction to energy (Δ*Ha*, 298 K), potential barriers with thermal correction to enthalpy (Δ*Eb*) and the reaction heats with thermal correction to enthalpy (Δ*Hb*, 298 K), and potential barriers with thermal correction to Gibbs Free energy (Δ*Ec*) and the reaction heats with thermal correction to Gibbs Free energy (Δ*Hc*, 298 K) of the formations of PCN from 2-CP at the MPWB1K/aug-cc-pVTZ//MPWB1K/6-31+G(d,p) level. Figures calculated at the MPWB1K/6-311+G(3df,2p)//MPWB1K/6-31+G(d,p) level. Supplementary materials can be found at http://www.mdpi.com/1422-0067/16/10/25641/s1.
