# Supplementary material for: Theoretical Mechanistic and Kinetic Studies on Homogeneous Gas-Phase Formation of Polychlorinated Naphthalene from 2-Chlorophenol as Forerunner
Source: Int J Mol Sci. 2015 Oct 26;16(10):25641–56. doi: 10.3390/ijms161025641 (PMC4632819; doi:10.3390/ijms161025641)
Supplement: Supplementary file 1 [file ijms-16-25641-s001.pdf]

Supplementary Information

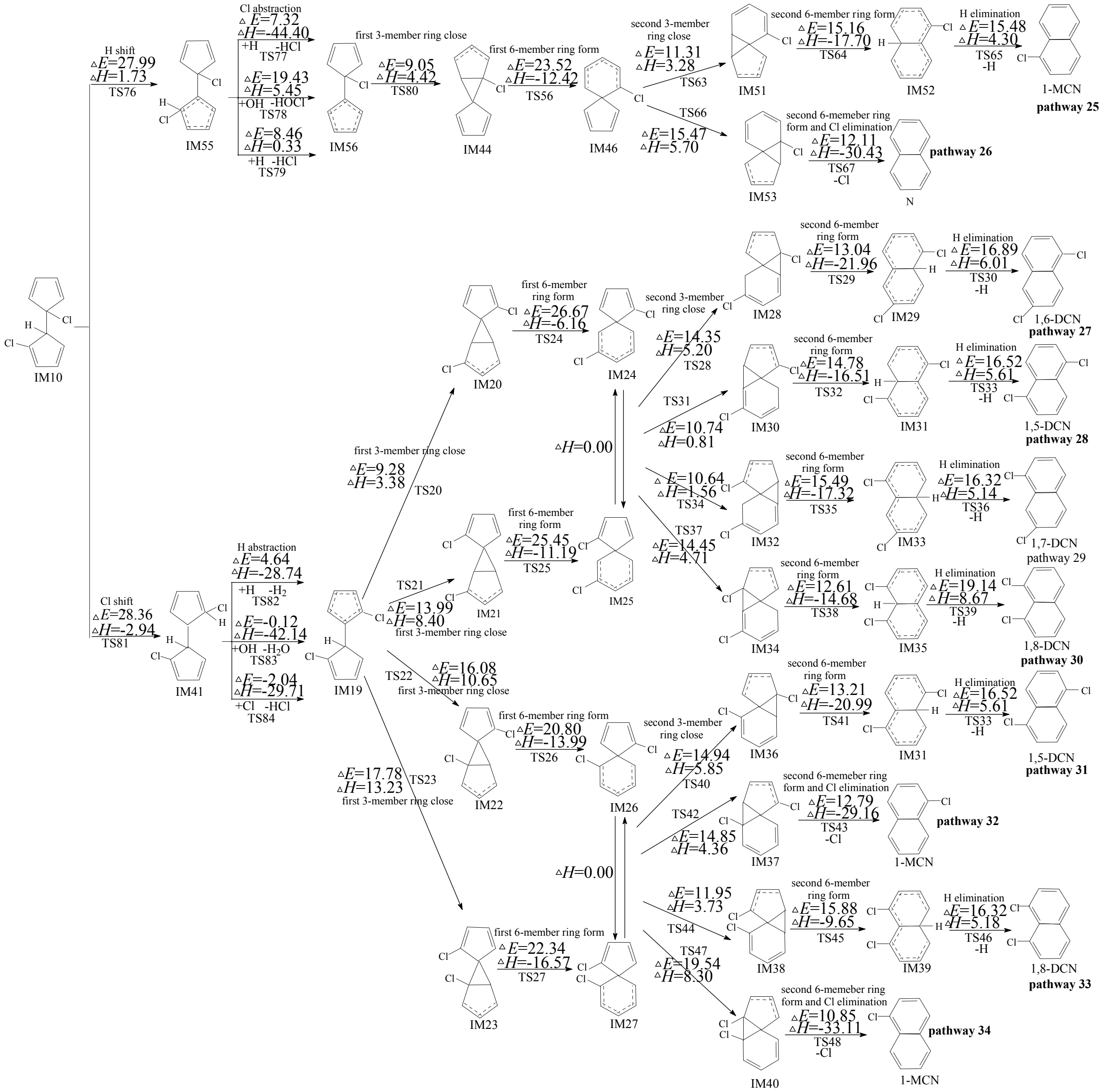

Figure S1. PCN formation routes from IM10 starting with H-shift step. These routes are embedded with the potential barriers Δ*E* (in kcal/mol) and reaction heats Δ*H* (in kcal/mol) at the MPWB1K/aug-cc-pVTZ//MPWB1K/6-31+G(d,p) level. Δ*H* is calculated at 0 K.

Table S1. Imaginary frequencies (in cm^{-1}), total energies (in a.u.), zero point energies (ZPE, in a.u.), thermal correction to energy (in a.u.), thermal correction to enthalpy (in a.u.), thermal correction to gibbs free energy (in a.u.), and for the transition states involved in the formation of PCNs from the 2-CP as precursor at the MPWB1K/aug-cc-pVTZ//MPWB1K/6-31+G(d,p) level.

Transition States	Imaginary Frequencies	Total Energies	ZPE	Thermal Correction to Energy	Thermal Correction to Enthalpy	Thermal Correction to Gibbs Free Energy
TSA	180i	−1532.917627	0.172823	0.185973	0.186917	0.131781
TS1	396i	−1532.893579	0.170831	0.184780	0.185724	0.127355
TS2	729i	−1532.830081	0.169217	0.183253	0.184197	0.126281
TS3	406i	−1419.565994	0.160118	0.172399	0.173343	0.119553
TS4	616i	−1419.504607	0.158503	0.171125	0.172069	0.117709
TSB	58i	−1532.917295	0.172738	0.185745	0.186689	0.132739
TS5	704i	−1532.884155	0.170718	0.184465	0.185409	0.128973
TS6	640i	−1532.826383	0.168640	0.182653	0.183597	0.126783
TS7	450i	−1419.554413	0.159761	0.172005	0.172949	0.120162
TS8	619i	−1419.496987	0.158123	0.170634	0.171578	0.118440
TS9	637i	−1532.863404	0.170475	0.184406	0.185350	0.128319
TS10	621i	−1532.823866	0.168744	0.182988	0.183932	0.126579
TS11	711i	−1419.558084	0.159503	0.171675	0.172619	0.119758
TS12	653i	−1419.496872	0.157662	0.170146	0.171090	0.117615
TSC	218i	−1532.913843	0.172426	0.185405	0.186350	0.133101
TS13	705i	−1532.871065	0.170122	0.183941	0.184885	0.128906
TS14	593i	−1532.816203	0.168213	0.182361	0.183305	0.126515
TS15	707i	−1419.545500	0.159381	0.171496	0.172440	0.120558
TS16	707i	−1419.494358	0.157761	0.170150	0.171094	0.118757
TS17	1323i	−1306.789118	0.150238	0.161318	0.162262	0.111709
TS18	528i	−1382.031285	0.160393	0.172588	0.173532	0.120361
TS19	528i	−1766.523083	0.148371	0.160475	0.161419	0.106457
TS20	460i	−1305.659410	0.138522	0.148191	0.149135	0.101425
TS21	481i	−1305.652030	0.138633	0.148197	0.149141	0.101942
TS22	543i	−1305.648354	0.138299	0.147889	0.148833	0.101787
TS23	477i	−1305.645264	0.137913	0.147584	0.148528	0.101195

Table S1. *Cont.*

Transition States	Imaginary Frequencies	Total Energies	ZPE	Thermal Correction to Energy	Thermal Correction to Enthalpy	Thermal Correction to Gibbs Free Energy
TS24	680i	−1305.625698	0.137907	0.147649	0.148594	0.101029
TS25	714i	−1305.619721	0.137989	0.147722	0.148667	0.101116
TS26	745i	−1305.620687	0.138301	0.147951	0.148895	0.101973
TS27	764i	−1305.617178	0.138180	0.147891	0.148835	0.101612
TS28	677i	−1305.655491	0.138253	0.147617	0.148561	0.102361
TS29	724i	−1305.649864	0.138811	0.148190	0.149134	0.102725
TS30	1166i	−1305.673835	0.133931	0.143515	0.144459	0.097624
TS31	607i	−1305.661595	0.138602	0.148068	0.149012	0.102211
TS32	778i	−1305.654351	0.139076	0.148545	0.149489	0.102640
TS33	1172i	−1305.672831	0.134023	0.143586	0.144530	0.097818
TS34	605i	−1305.661656	0.138505	0.147955	0.148899	0.102126
TS35	796i	−1305.651904	0.138971	0.148444	0.149388	0.102552
TS36	1183i	−1305.673084	0.133871	0.143450	0.144395	0.097595
TS37	676i	−1305.655471	0.138380	0.147735	0.148679	0.102535
TS38	708i	−1305.651475	0.138950	0.148348	0.149292	0.102851
TS39	1150i	−1305.659294	0.133794	0.143485	0.144429	0.096302
TS40	684i	−1305.655798	0.138594	0.147934	0.148878	0.102735
TS41	719i	−1305.649456	0.138816	0.148180	0.149124	0.102879
TS42	647i	−1305.655979	0.138631	0.148090	0.149034	0.102542
TS43	721i	−1305.652548	0.138852	0.148318	0.149262	0.102805
TS44	638i	−1305.660783	0.138811	0.148223	0.149167	0.102670
TS45	786i	−1305.648558	0.138786	0.148292	0.149236	0.102462
TS46	1181i	−1305.658124	0.133683	0.143355	0.144299	0.096820
TS47	708i	−1305.648423	0.138548	0.147863	0.148807	0.103085
TS48	650i	−1305.648912	0.138430	0.147944	0.148888	0.102535
TS49	1208i	−1306.256613	0.149141	0.159013	0.159957	0.112312
TS50	1031i	−1306.787314	0.153246	0.164844	0.165788	0.113775
TS51	636i	−1382.002760	0.163907	0.176262	0.177206	0.123515

Table S1. *Cont.*

Transition States	Imaginary Frequencies	Total Energies	ZPE	Thermal Correction to Energy	Thermal Correction to Enthalpy	Thermal Correction to Gibbs Free Energy
TS52	521i	−1766.500840	0.150630	0.163028	0.163972	0.108524
TS53	521i	−845.970214	0.147958	0.156439	0.157383	0.112934
TS54	490i	−845.964317	0.147597	0.156056	0.157000	0.113054
TS55	686i	−845.936515	0.147476	0.155998	0.156942	0.112734
TS56	693i	−845.933627	0.147021	0.155599	0.156543	0.112496
TS57	602i	−845.973595	0.147911	0.156124	0.157068	0.113727
TS58	797i	−845.963093	0.148272	0.156519	0.157463	0.114032
TS59	1181i	−845.986658	0.143769	0.152131	0.153075	0.109543
TS60	604i	−845.973528	0.147975	0.156204	0.157148	0.113768
TS61	780i	−845.965495	0.148495	0.156718	0.157662	0.114274
TS62	1166i	−845.986421	0.143658	0.152038	0.152982	0.109448
TS63	617i	−845.974016	0.148161	0.156356	0.157300	0.114011
TS64	801i	−845.962672	0.148160	0.156414	0.157358	0.113998
TS65	1185i	−845.985728	0.143527	0.151919	0.152863	0.109329
TS66	652i	−845.967282	0.148046	0.156256	0.157200	0.114143
TS67	723i	−845.963708	0.148210	0.156447	0.157391	0.114337
TS68	996i	−1306.784507	0.153052	0.164618	0.165562	0.114217
TS69	587i	−1382.000890	0.163024	0.175765	0.176709	0.121959
TS70	397i	−1766.500424	0.150369	0.162861	0.163805	0.108505
TS71	1402i	−1306.783315	0.149941	0.161043	0.161987	0.111576
TS72	665i	−1382.026268	0.159666	0.171893	0.172838	0.119749
TS73	47i	−1766.515143	0.148375	0.161180	0.162124	0.108625
TS74	468i	−1305.648056	0.138157	0.147776	0.148720	0.101761

Table S1. *Cont.*

Transition States	Imaginary Frequencies	Total Energies	ZPE	Thermal Correction to Energy	Thermal Correction to Enthalpy	Thermal Correction to Gibbs Free Energy
TS75	477i	−1305.645264	0.137915	0.147585	0.148529	0.101200
TS76	1253i	−1306.247324	0.148559	0.158577	0.159521	0.111644
TS77	1040i	−1306.779648	0.152783	0.164489	0.165434	0.113072
TS78	653i	−1381.994699	0.162817	0.175526	0.176470	0.121708
TS79	486i	−1766.496033	0.150462	0.162819	0.163763	0.108778
TS80	490i	−845.964314	0.147597	0.156055	0.156999	0.113055
TS81	615i	−1306.248919	0.150738	0.160747	0.161691	0.113892
TS82	1463i	−1306.789060	0.150494	0.161556	0.162500	0.111562
TS83	1463i	−1382.030541	0.160064	0.172328	0.173273	0.119349
TS84	84i	−1766.519541	0.149810	0.161530	0.162475	0.108876

Table S2. The potential barriers with ZPE correction (Δ*E*) and the reaction heats with ZPE correction (Δ*H*, 0 K), potential barriers with thermal correction to energy (Δ*Ea*) and the reaction heats with thermal correction to energy (Δ*Ha*, 298 K), potential barriers with thermal correction to enthalpy (Δ*Eb*) and the reaction heats with thermal correction to enthalpy (Δ*Hb*, 298 K), and potential barriers with thermal correction to Gibbs Free energy (Δ*Ec*) and the reaction heats with thermal correction to Gibbs Free energy (Δ*Hc*, 298 K) of the formations of PCN from 2-CP at the MPWB1K/aug-cc-pVTZ//MPWB1K/6-31+G(d,p) level.

TS	Δ*E*	Δ*H*	Δ*Ea*	Δ*Ha*	Δ*Eb*	Δ*Hb*	Δ*Ec*	Δ*Hc*
TSA	9.68	−16.63	10.04	−15.98	9.44	−16.57	23.67	−3.96
TS1	40.15	36.15	40.36	37.02	40.36	37.02	39.94	34.44
TS2	42.83	−26.05	42.22	−26.51	42.22	−25.92	44.68	−34.50
TS3	40.48	36.45	40.71	37.27	40.71	37.27	40.26	34.71
TS4	41.54	−22.14	41.16	−22.66	41.16	−22.07	42.91	−30.12
TSB	9.84	−8.62	10.10	−7.95	9.51	−8.54	24.48	4.83
TS5	37.98	21.21	38.05	21.97	38.05	21.97	38.08	19.44
TS6	51.72	−9.61	51.19	−10.00	51.19	−9.41	53.52	−18.10
TS7	38.01	31.06	38.24	31.97	38.24	31.97	37.72	28.79
TS8	41.96	−23.77	41.45	−24.20	41.45	−23.61	43.89	−32.26
TSB	9.84	−8.62	10.10	−7.95	9.51	−8.54	24.48	4.83
TS9	50.85	31.99	51.03	32.80	51.03	32.80	50.69	30.20
TS10	42.58	−24.17	42.15	−24.56	42.15	−23.97	44.21	−32.87
TS11	39.32	22.56	39.47	23.23	39.47	23.23	39.17	21.62
TS12	54.02	−11.50	53.69	−11.73	53.69	−11.14	54.62	−21.08
TSC	11.81	0.45	12.05	1.08	11.46	0.49	26.88	14.46
TS13	36.76	17.72	36.90	18.42	36.90	18.42	36.62	16.36
TS14	52.26	−11.22	51.92	−11.54	51.92	−10.95	53.19	−20.22
TS15	39.40	18.90	39.58	19.63	39.58	19.63	39.13	17.59
TS16	51.57	−12.84	51.19	−13.06	51.19	−12.47	52.50	−22.28
TS17	3.99	−29.19	3.76	−28.50	3.17	−28.50	9.45	−30.38

Table S2. *Cont.*

TS	Δ*E*	Δ*H*	Δ*Ea*	Δ*Ha*	Δ*Eb*	Δ*Hb*	Δ*Ec*	Δ*Hc*
TS18	−0.84	−42.59	−0.96	−42.20	−1.55	−42.20	7.61	−43.70
TS19	−5.62	−30.16	−5.20	−29.47	−5.79	−29.47	0.88	−32.21
TS20	9.28	3.38	8.96	3.09	8.96	3.09	9.63	3.84
TS21	13.99	8.40	13.60	8.12	13.60	8.12	14.59	8.88
TS22	16.08	10.65	15.71	10.35	15.71	10.35	16.80	11.49
TS23	17.78	13.23	17.46	12.96	17.46	12.96	18.36	13.96
TS24	26.67	−6.16	26.68	−5.94	26.68	−5.94	26.69	−6.58
TS25	25.45	−11.19	25.46	−10.97	25.46	−10.97	25.46	−11.62
TS26	22.80	−13.99	22.75	−13.79	22.75	−13.79	22.78	−14.59
TS27	22.34	−16.57	22.31	−16.40	22.31	−16.40	22.29	−17.04
TS28	14.35	5.20	13.91	4.83	13.91	4.83	15.42	6.25
TS29	13.04	−21.96	12.96	−21.82	12.96	−21.82	12.92	−22.60
TS30	16.89	6.01	16.81	6.51	16.81	7.10	17.28	0.34
TS31	10.74	0.81	10.36	0.52	10.36	0.52	11.49	1.45
TS32	14.78	−16.51	14.68	−16.47	14.68	−16.47	14.86	−16.67
TS33	16.52	5.61	16.44	6.10	16.44	6.69	16.90	−0.07
TS34	10.64	1.56	10.25	1.27	10.25	1.27	11.40	2.27
TS35	15.49	−17.32	15.41	−17.25	15.41	−17.25	15.51	−17.54
TS36	16.32	5.14	16.23	5.62	16.23	6.21	16.65	−0.60
TS37	14.45	4.71	13.99	4.32	13.99	4.32	15.54	5.78
TS38	12.61	−14.68	12.57	−14.59	12.57	−14.59	12.46	−15.04
TS39	19.14	8.67	19.21	9.30	19.21	9.90	18.49	2.35
TS40	14.94	5.85	14.50	5.51	14.50	5.51	15.82	6.72
TS41	13.21	−20.99	13.13	−20.86	13.13	−20.86	13.16	−21.58
TS42	14.85	4.36	14.49	4.06	14.49	4.06	15.58	5.11
TS43	12.79	−29.16	12.73	−29.33	12.73	−28.74	12.79	−37.22
TS44	11.95	3.73	11.56	3.45	11.56	3.45	12.65	4.31
TS45	15.88	−9.65	15.82	−10.08	15.82	−10.08	15.88	−8.99
TS46	16.32	5.18	16.80	6.25	16.80	6.84	15.33	−1.87
TS47	19.54	8.30	19.09	7.97	19.09	7.97	20.67	9.43
TS48	10.85	−33.11	10.86	−33.25	10.86	−32.65	10.59	−41.53
TS49	25.01	−0.45	24.91	−0.26	24.91	−0.26	24.86	−1.27
TS50	7.46	−43.53	7.36	−43.61	6.77	−43.61	13.15	−47.15
TS51	19.72	6.32	19.51	6.00	18.91	6.00	28.76	3.47
TS52	10.21	1.21	10.62	1.23	10.02	1.23	17.40	−1.71
TS53	9.36	4.70	8.88	4.25	8.88	4.25	10.25	5.70
TS54	12.84	8.21	12.34	7.80	12.34	7.80	14.02	9.41
TS55	25.51	−8.54	25.49	−8.34	25.49	−8.34	25.57	−8.88
TS56	23.52	−12.42	23.51	−12.28	23.50	−12.28	23.52	−12.81
TS57	11.05	3.01	10.64	2.69	10.64	2.69	11.81	3.77
TS58	14.86	−18.69	14.78	−18.60	14.78	−18.60	14.82	−19.03
TS59	15.93	4.82	15.84	5.30	15.84	5.89	16.24	−0.95
TS60	11.13	2.29	10.73	1.99	10.73	1.99	11.87	3.00

Table S2. *Cont.*

TS	Δ*E*	Δ*H*	Δ*Ea*	Δ*Ha*	Δ*Eb*	Δ*Hb*	Δ*Ec*	Δ*Hc*
TS61	14.20	−18.02	14.11	−17.95	14.11	−17.95	14.23	−18.30
TS62	16.06	5.23	15.98	5.73	15.98	6.32	16.38	−0.50
TS63	11.31	3.28	10.92	2.97	10.92	2.97	11.93	3.90
TS64	15.16	−17.70	15.10	−17.59	15.10	−17.59	15.15	−18.01
TS65	15.48	4.30	15.40	4.79	15.40	5.38	15.76	−1.49
TS66	15.47	5.70	15.08	5.40	15.08	5.40	16.24	6.50
TS67	12.11	−30.43	12.04	−30.58	12.04	−29.99	12.11	−38.56
TS68	6.16	−46.47	6.08	−46.51	5.49	−46.51	11.79	−50.54
TS69	17.40	3.39	17.47	3.10	16.87	3.10	25.56	0.08
TS70	7.37	−1.73	7.87	−1.67	7.28	−1.67	14.25	−5.11
TS71	4.96	−26.47	4.58	−25.87	3.99	−25.87	10.88	−27.18
TS72	−0.63	−39.87	−0.89	−39.56	−1.48	−39.56	8.25	−40.51
TS73	−3.11	−27.44	−2.42	−26.84	−3.01	−26.84	5.09	−29.01
TS74	10.97	5.44	10.55	5.08	10.55	5.08	11.65	6.17
TS75	12.57	8.02	12.18	7.69	12.18	7.69	13.05	8.63
TS76	27.99	1.73	27.82	1.79	27.82	1.79	28.14	1.30
TS77	7.32	−44.40	7.26	−44.55	6.66	−44.55	12.82	−47.76
TS78	19.43	5.45	19.41	5.06	18.82	5.06	27.99	2.86
TS79	8.46	0.33	8.81	0.30	8.22	0.30	15.88	−2.32
TS80	9.05	4.42	8.58	4.04	8.58	4.04	9.94	5.33
TS81	28.36	−2.94	28.19	−2.90	28.19	-2.90	28.55	−3.39
TS82	4.64	−28.74	4.20	−28.24	3.61	−28.24	10.66	−29.11
TS83	−0.12	−42.14	−0.40	−41.94	−0.99	−41.94	8.71	−42.44
TS84	−2.04	−29.71	−2.06	−29.21	−2.65	−29.21	5.88	−30.94

Table S3. The potential barriers (Δ*E*) and the reaction heats (Δ*H*) of the formations of 2-CPRs from 2-CP were calculated at the MPWB1K/6-311+G(3df,2p)//MPWB1K/6-31+G(d,p) level.

Reaction	Δ*E*	Δ*H*	Reference
2-CP → 2-CPR + H	0.00	85.91	[34]
2-CP + H → 2-CPR + H_2	13.80	−12.01	[34]
2-CP + OH → 2-CPR + H_2O	3.20	−26.91	[35]
2-CP + O(^{3}P) → 2-CPR + OH	7.52	−11.34	this study
2-CP + Cl → 2-CPR + HCl	−2.32	−14.96	this study

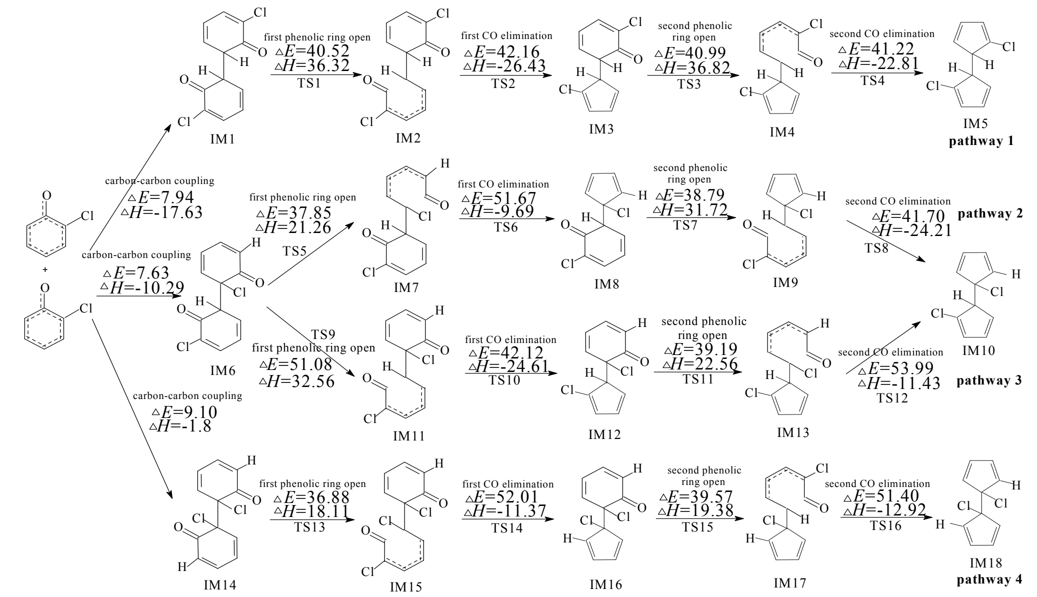

Figure S2. Chlorinated dihydrofulvalene formation routes embedded with the potential barriers Δ*E* (in kcal/mol) and reaction heats Δ*H* (in kcal/mol) from the 2-CP as precursor at the MPWB1K/6-311+G(3df,2p)//MPWB1K/6-31+G(d,p) level. Δ*H* is calculated at 0 K.

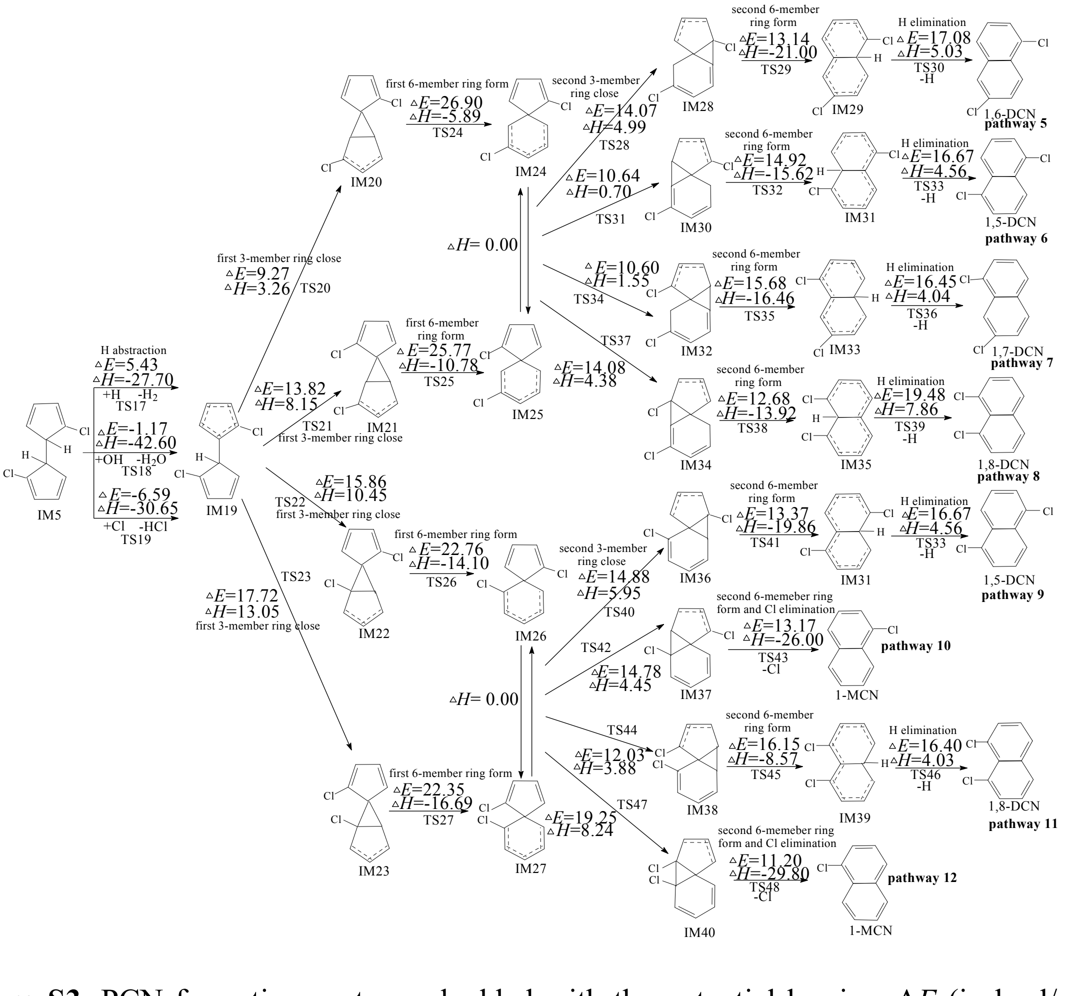

Figure S3. PCN formation routes embedded with the potential barriers Δ*E* (in kcal/mol) and reaction heats Δ*H* (in kcal/mol) from IM5 at the MPWB1K/6-311+G(3df,2p)// MPWB1K/6-31+G(d,p) level. Δ*H* is calculated at 0 K.

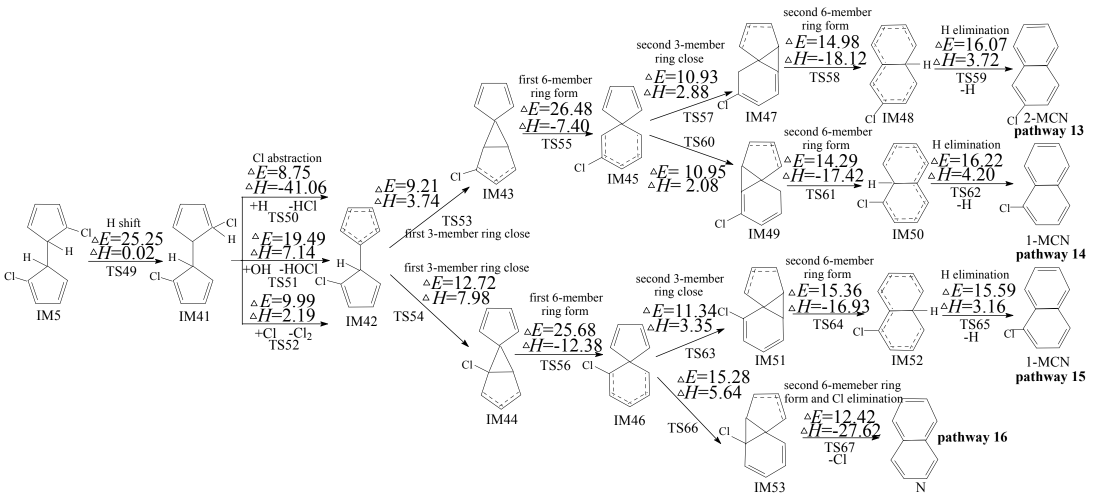

Figure S4. PCN formation routes from IM5 proposed by Kim [21,22], starting with H-shift step. These routes are embedded with the potential barriers Δ*E* (in kcal/mol) and reaction heats Δ*H* (in kcal/mol) at the MPWB1K/6-311+G(3df,2p)//MPWB1K/6-31+G(d,p) level. Δ*H* is calculated at 0 K.

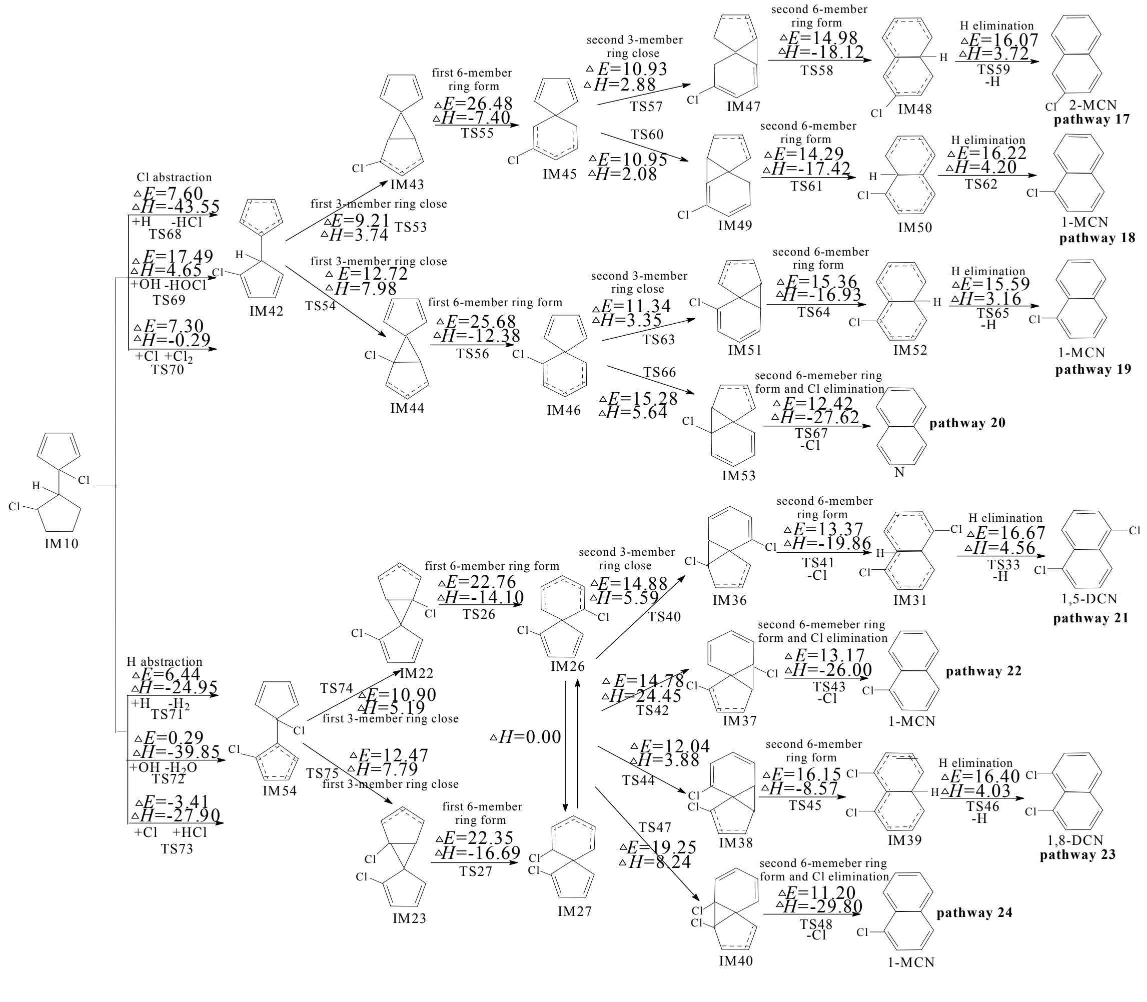

Figure S5. PCN formation routes embedded with the potential barriers Δ*E* (in kcal/mol) and reaction heats Δ*H* (in kcal/mol) from IM10 at the MPWB1K/6-311+G(3df,2p)// MPWB1K/6-31+G(d,p) level. Δ*H* is calculated at 0 K.

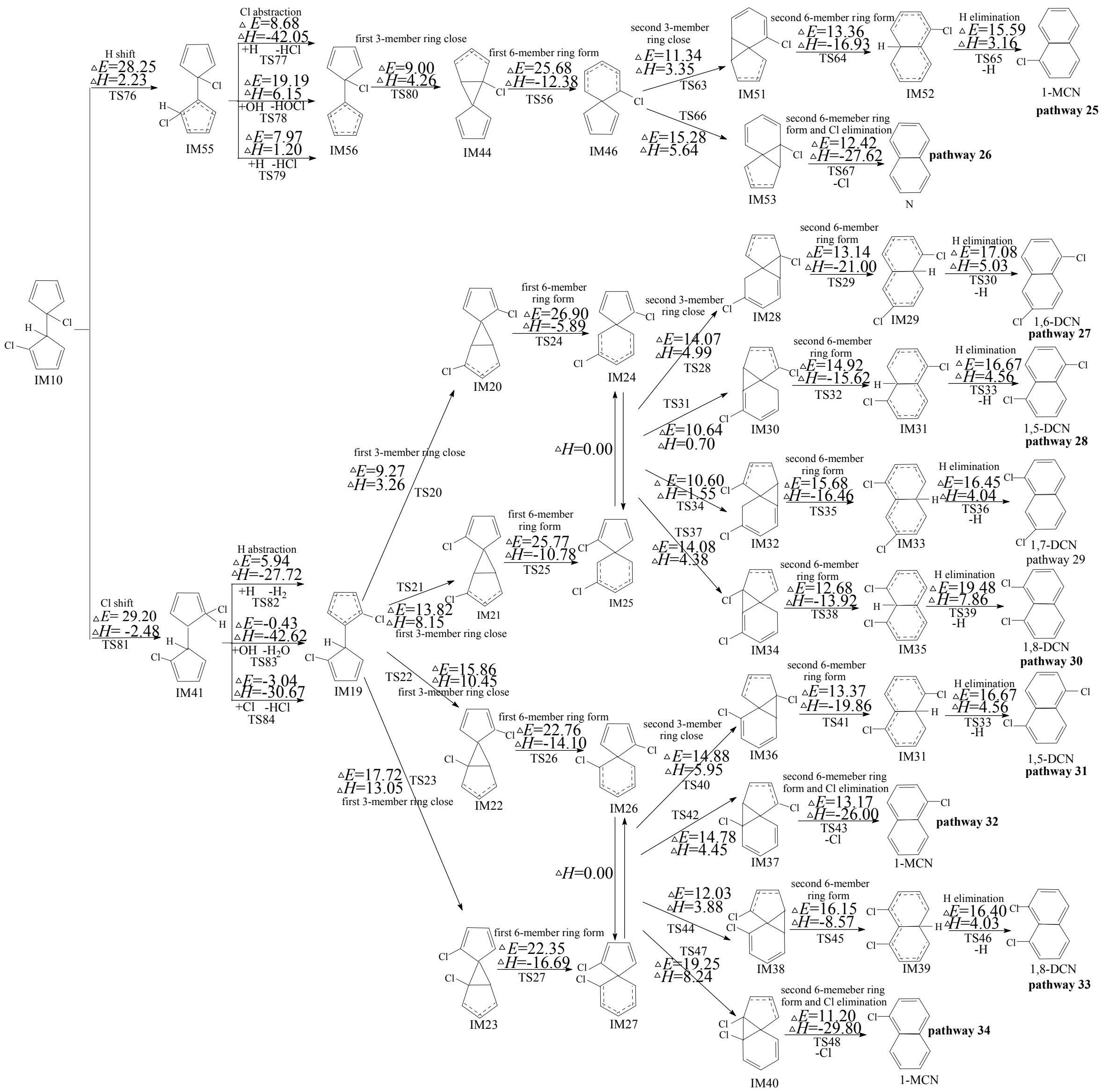

Figure S6. PCN formation routes from IM10 starting with H-shift step. These routes are embedded with the potential barriers Δ*E* (in kcal/mol) and reaction heats Δ*H* (in kcal/mol) at the MPWB1K/6-311+G(3df,2p)//MPWB1K/6-31+G(d,p) level. Δ*H* is calculated at 0 K.
